# MamA as a Model Protein for Structure-Based Insight into the Evolutionary Origins of Magnetotactic Bacteria

**DOI:** 10.1371/journal.pone.0130394

**Published:** 2015-06-26

**Authors:** Natalie Zeytuni, Samuel Cronin, Christopher T. Lefèvre, Pascal Arnoux, Dror Baran, Zvi Shtein, Geula Davidov, Raz Zarivach

**Affiliations:** 1 Department of Life Sciences and The National Institute for Biotechnology in the Negev, Ben-Gurion University of the Negev, Beer Sheva, Israel; 2 CEA/CNRS/Aix-Marseille Université, UMR 7265 Biologie Végétale et Microbiologie Environnementales, Laboratoire de Bioénergétique Cellulaire, Saint Paul les Durance, France; Louisiana State University and A & M College, UNITED STATES

## Abstract

MamA is a highly conserved protein found in magnetotactic bacteria (MTB), a diverse group of prokaryotes capable of navigating according to magnetic fields – an ability known as magnetotaxis. Questions surround the acquisition of this magnetic navigation ability; namely, whether it arose through horizontal or vertical gene transfer. Though its exact function is unknown, MamA surrounds the magnetosome, the magnetic organelle embedding a biomineralised nanoparticle and responsible for magnetotaxis. Several structures for MamA from a variety of species have been determined and show a high degree of structural similarity. By determining the structure of MamA from *Desulfovibrio magneticus* RS-1 using X-ray crystallography, we have opened up the structure-sequence landscape. As such, this allows us to perform structural- and phylogenetic-based analyses using a variety of previously determined MamA from a diverse range of MTB species across various phylogenetic groups. We found that MamA has remained remarkably constant throughout evolution with minimal change between different taxa despite sequence variations. These findings, coupled with the generation of phylogenetic trees using both amino acid sequences and 16S rRNA, indicate that magnetotaxis likely did not spread via horizontal gene transfer and instead has a significantly earlier, primordial origin.

## Introduction

Magnetotactic bacteria (MTB) are a diverse group of prokaryotes capable of movement according to magnetic fields–an ability known as magnetotaxis. This characteristic is believed to have developed to enable these aerobic or anaerobic, aquatic, Gram negative bacteria to optimally find the oxic-anoxic boundary [1]. This is achieved via the internal biomineralisation of magnetic nanoparticles of either magnetite (Fe_3_O_4_) or greigite (Fe_3_S_4_) by a specific organelle, the magnetosome. Each of these membrane-bound organelles biomineralises a single magnetic crystal, following which the organelles are aligned into a chain, creating a magnetic moment [2].

First brought to wide scientific attention in 1975, approximately 30 species of MTB have been studied and their genomes sequenced [3,4]. Until the early 90s, it was thought that MTB were restricted to the *Alphaproteobacteria* class. With the discovery of the multicellular magnetotactic prokaryotes [5] and *Desulfovibrio magneticus* strain RS-1 [6] the diversity of MTB extended to the *Deltaproteobacteria* class. Later, magnetotactic bacteria were found in three other major groups of the prokaryotes: the *Nitrospirae* [7], the *Gammaproteobacteria* class [8] and the OP3 division [9]. *Desulfovibrio magneticus* RS-1, which shares 98.7% similarity of its 16S rRNA gene sequence with *Desulfovibrio burkinensis*, is a magnetite-containing microorganism related to dissimilatory sulphate-reducing bacteria [10].

Many of the magnetosome-associated genes are found on a highly conserved genomic cluster known as the *magnetosome island* [1]. In particular, the protein MamA is highly conserved amongst MTB [11]. Considerable research has focused on the origins of magnetotaxis; that is, whether there is a monophyletic origin for this ability or whether it was acquired through horizontal gene transfer (HGT) [12,13]. Of the magnetosome genes, studies have found these either to be essential, in that deletion results in a noticeable change or loss of function, or inessential; research has found that these inessential genes are easily lost during magnetosome evolution. MamA, on the other hand, unexpectedly is neither–deletion of the *mamA* genes results in no significant change in function in cultivated cells, yet MamA is almost perfectly conserved and is rarely lost [14,15]. Indeed, *mamA* belongs to the nine “core genes” essential for magnetosome formation [16,17], though it has yet to be determined specifically how it is essential. *Nitrospirae* and *Deltaproteobacteria* represent two of the most deeply branching groups of MTB and, along with the *Alphaproteobacteria*, all of these MTB carry in their genome a copy of *mamA*; this protein is therefore a prime candidate for comparative structural studies determining protein conservation between different, distantly related species, as well as for attempting to illuminate on the origins of MTB.

The hypothesis for the earlier, primordial development of magnetotaxis–as opposed to its acquisition via HGT–is further supported by the physical shape of the magnetic nanocrystals; the magnetosomes of *D*. *magneticus* RS-1 (hereafter, RS-1) synthesise anisotropic, bullet-shaped magnetite particles, similar to those produced by the known MTB of the *Nitrospirae*, “*Candidatus* Magnetobacterium bavaricum”, (hereafter, Mbav). Conversely, MTB of the much later-diverging *Alpha*- and *Gammaproteobacteria* will only produce well-defined, cuboctahedral crystals [13].

MamA is a magnetosome-associated protein (MAP) involved in the biomineralisation process by coating the magnetosome membrane [18]. It has previously been genetically manipulated and the structures of its ∆41 mutants–created by removing the putative N-terminal TPR repeat–solved in multiple species of MTB, mostly *Alphaproteobacteria* (*Magnetospirillum magneticum* AMB-1, PDB code: 3AS5; *Magnetospirillum gryphiswaldense* MSR-1, PDB code: 3AS8; and Mbav, PDB code: 3VTX) [11,19]. This 23 kDa protein is comprised of five tetratrico-peptide repeats (TPR), a 34 amino acid structural motif. Each TPR consists of a helix-turn-helix fold, in addition to a putative N-terminal repeat. As such, MamA adopts an overall superhelical structure enabling the protein to be highly involved in protein-protein interactions via two main binding sites, one at its concave surface and the other at its convex surface [19].

Studies have been performed using MamA from Mbav (from the *Nitrospirae* phylum) and from the *Magnetospirillum* genus, which represent the evolutionary/phylogenetic ends of MTB; by choosing RS-1 as our model organism we provide here a full spread across the evolutionary landscape, with a magnetotactic *Deltaproteobacteria* between the two extremes. Here, we have solved a new structure for MamA from the alternative species, *Desulfovibrio magneticus* strain RS-1, and compare between other, previously solved MamA structures and this distantly related species of MTB. Comparative structural analysis gives an insight into the evolution of magnetotaxis.

## Results and Discussion

### MamAΔ41 purification and crystallisation

To obtain biochemical and structural information toward comparative studies, recombinant MamAΔ41_RS-1_ was overexpressed in *Escherichia coli* cells. MamAΔ41RS-1 was found to be soluble and stable in a buffer solution containing high NaCl concentration (> 0.5 M), whereas previously characterised MamAΔ41 from different species were soluble at lower NaCl concentrations (~150 mM) [11,19–21]. Size-exclusion chromatography revealed that MamAΔ41_RS-1_ elutes at a volume that corresponds to an octamer, whilst MamAΔ41 proteins from different species were found as either trimers or monomers (S1 Fig). The purified MamAΔ41_RS-1_ was subjected to numerous crystallisation trials using a sitting drop vapour diffusion methodology. Those crystallisation trails did not result in any crystal nucleation, even after subjecting the protein to lysine methylation and limited proteolysis. To overcome this crystallisation obstacle we utilised Surface Entropy Reduction (SER), which suggests replacing surface-exposed high entropy amino acids with residues that have small, low entropy side chains, such as alanines. Residues of particular importance are lysines and glutamates as statistical analyses show that both are localised predominantly on the surface [22] and are disfavoured at protein-protein interfaces [23]. We submitted the MamAΔ41_RS-1_ protein sequence to the SERp web-server (http://services.mbi.ucla.edu/SER/) [24] and followed its suggestion to mutate three surface predicted residues–Glu140, Lys141 and Glu143 –to alanines. As such, the triple mutated protein, named MamAΔ41 RS-1 TM (henceforth, ArsTM), was overexpressed and purified in the same manner as the wild-type MamAΔ41_RS-1_. The size-exclusion chromatography elution profile of ArsTM displayed some differences in reference to MamAΔ41_RS-1_, as the mutated protein eluted at three distinct volumes. By comparing these volumes to a standard calibration curve we determined that the three peaks correspond to sizes of a 13-meric oligomer, an octamer and a monomer (S1 Fig). Crystallisation trials of octameric and monomeric ArsTM resulted in the appearance of a single crystal form that diffracted to a resolution of 2.88 Å (see Table 1 for data collection and refinement statistics). Phase information was obtained by the molecular replacement technique and, after rounds of manual rebuilding and refinement, we obtained a high quality structure (Table 1) with almost a full cover of the protein sequence (S1 Table).

**Table 1:** Crystallographic data for ArsTM.

PDB code	4XI0
Protein	MamAΔ41
**Data collection**	ID14-4 - ESRF
Space group	I_4_
Cell dimensions	
*a*, *b*, *c* (Å)	151.087, 151.087, 204.903
α, β, γ (°)	90, 90, 90
Resolution (Å)	2.88
*R*sym or *R*merge	6.5 (60.8)
*I* / σ*I*	43.89 (3.64)
Completeness (%)	99.8 (100)
Redundancy	6.2
Wavelength (Å)	0.939
**Refinement**	
Resolution (Å)	2.88
No. reflections	51648
*R*work / *R*free	20.94/22.01
No. atoms	
Protein	8530
Ligand/ion	
Water	
*B*-factors	
Protein	77.52
Ligand/ion	
Water	
R.m.s. deviations	
Bond lengths (Å)	0.0237
Bond angles (°)	1.8972

### Phylogenetic analyses of MamA

MamA protein can be retrieved from 30 known MTB genomes. A total of 32 sequences could be found due to the presence of two copies of *mamA* in strains HK-1 [9] and BW-1 [17] that appear to have two clusters of *mam* genes, one for magnetite and the other for greigite magnetosome formation. All 32 MamA proteins contain five TPR domains except for strains UT-2, UT-4, LEMS, NML-1, SS-4, KR-1, CB-1, CC-2, LM-5, LM-2 and UT-1 whose C-terminal extremity remains incompletely sequenced. Using principal component analysis, we could not find a relation between the five TPR motifs that appear to have evolved from a common MamA-like ancestor that originally had the five TPR motifs (data not shown), rather than an evolution where one original TPR would have duplicated to give rise to the other TPR motifs. Although all 32 MamA proteins presumably have the same function in the different MTB based on their presence and synteny conservation in the *mamAB* operon between the different MTB from the different phylogenetic group, at first glance a BLAST search of a MamA does not retrieve the homologous proteins from MamA that are phylogenetically distant. For instance, MamA from magnetotactic *Alphaproteobacteria* are too divergent from the MamA of the magnetotactic *Deltaproteobacteria* (e.g. MamA of strain AMB-1 having only 24% identity with MamA of strain RS-1). Thus, although MamA appears to be well conserved in all MTB based on domain conservation, only a precise comparative genomic analysis allows finding homologues in other phylogenetically distant genomes.

**Fig 1:**
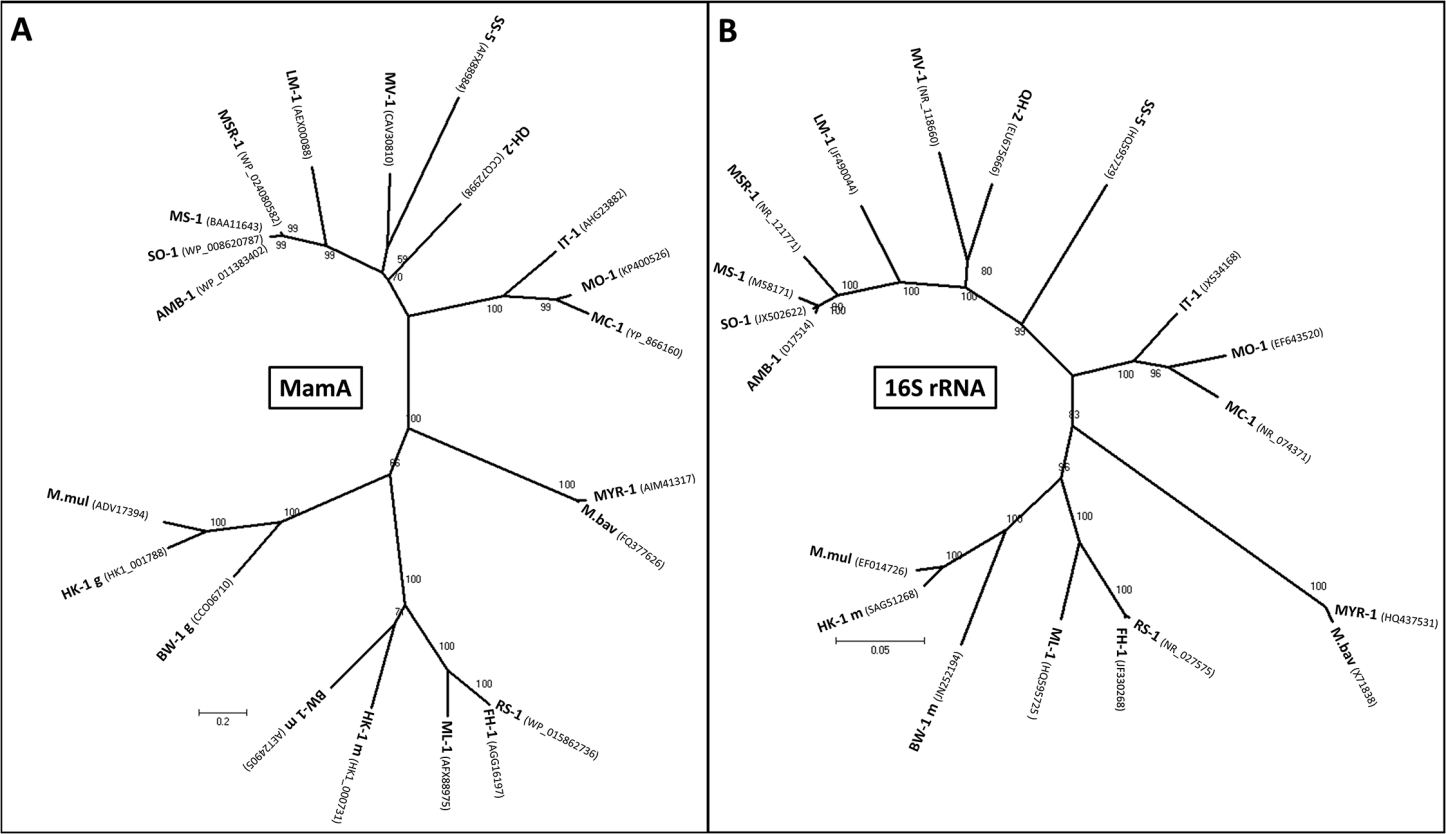
Congruency of the phylogenetic trees based on *(A)* MamA protein sequences and on *(B)* 16S rRNA gene sequences that reflect the evolution of MTB. Scale bars represent the percentage sequence divergence. Bootstrap values at nodes are percentages of 100 replicates. The MTB from *Alphaproteobacteria* class used in the analyses are: *Magnetospirillum magnetotacticum* (strain MS-1), *Ms*. *magneticum* (AMB-1), *Ms*. *gryphiswaldense* (MSR-1), strain SO-1, strain LM-1, *Magnetovibrio blakemorei* (MV-1), *Magnetospira* sp. QH-2, strain MO-1, *Magnetofaba australis* (IT-1) and *Magnetococcus marinus* (MC-1). Strain SS-5 from the *Gammaproteobacteria* class is also used. From the *Deltaproteobacteria* class MTB used include the magnetotactic multicellular prokaryotes *Ca*. Magnetoglobus multicellularis (MMP) and strain HK-1, *Ca*. Desulfamplus magnetomortis (BW-1), *Desulfovibrio magneticus* (RS-1 and FH-1), and strain ML-1. *Ca*. Magnetobacterium bavaricum (Mbav) and strain MYR-1 of the *Nitrospirae* phylum was also used. Accession numbers are shown in parenthesis.

We aligned the MamA amino acid sequences from the 21 complete MamA proteins (S2 Fig). Phylogenetic trees based on MamA amino acid sequences and 16S rRNA gene sequences show a congruency and a conservation of the different groups that contain MTB (Fig 1). This indicates that *mamA* evolved monophyletically, like the others magnetosome genes, from a common ancestor of all MTB.

**Fig 2:**
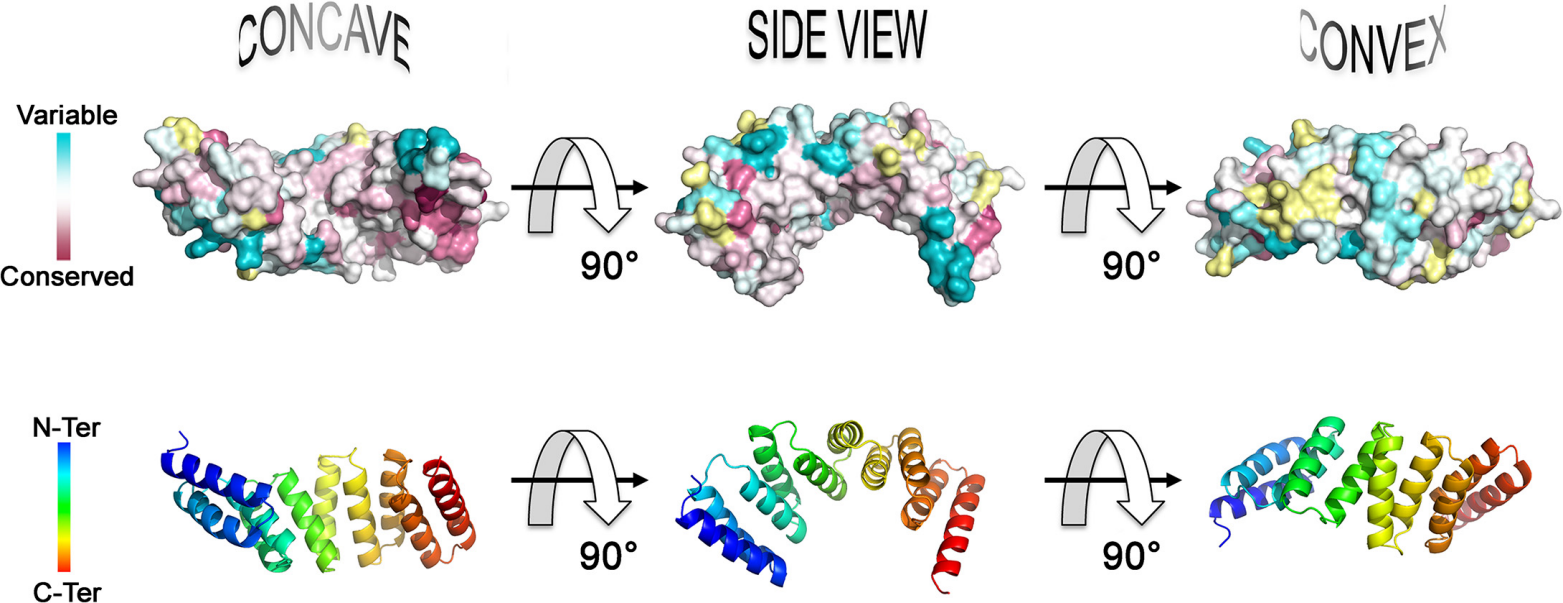
ConSurf analysis shows a high degree of structural conservation between ArsTM and MamA from alternative species, though this is largely limited to the concave surface and a cluster around the fifth TPR and the C-terminal. All sequences used in this analysis are shown in the multiple sequence alignment of S2 Fig.

These findings are reinforced by analysis using ConSurf [25,26], a tool for determining evolutionary conservation of amino acids using phylogenetics, which displayed a high degree of structural conservation between ArsTM and MamA from other species of MTB (Fig 2). In particular, the fifth TPR around the C-terminus is highly conserved, as well as the concave surface of the protein. Conversely, the backbone of the monomer is distinctly unconserved displaying only a few small conserved patches that are clusters of single or double residues.

### ArsTM crystal packing

**Fig 3:**
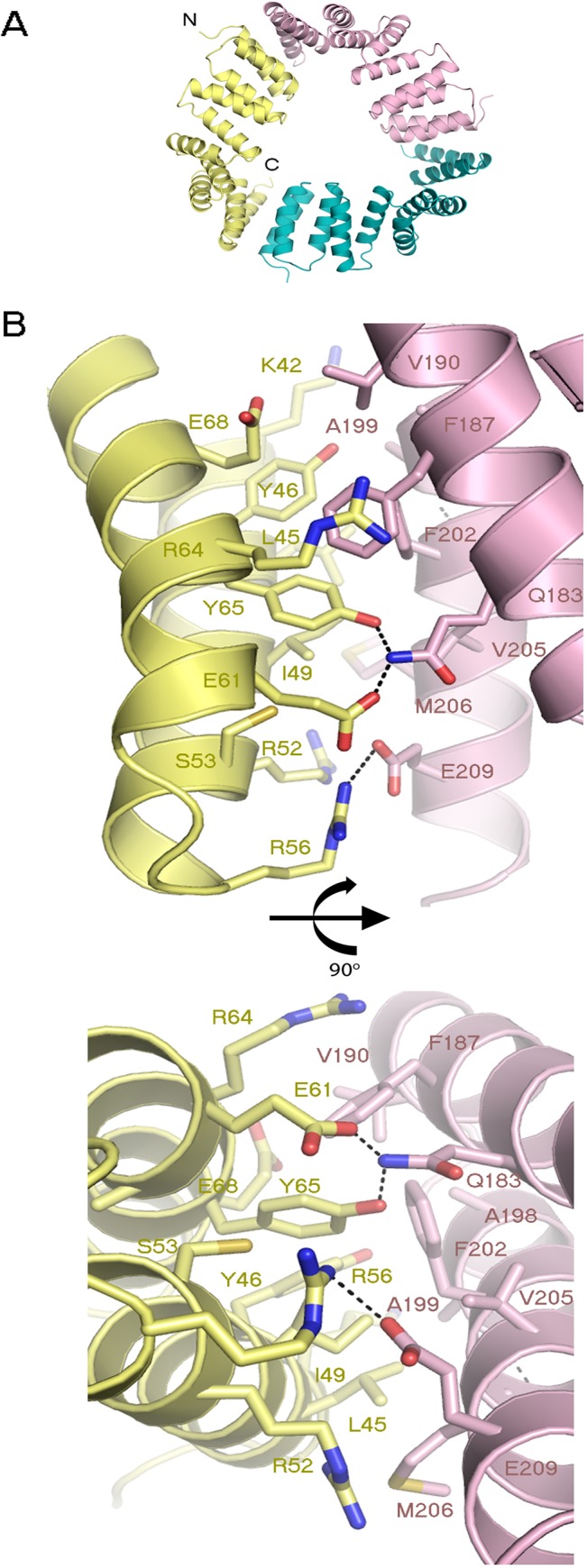
*(A)* Three ArsTM monomers form the trimeric ring. This flat ring encloses a ~15 Å diameter inner void. ***(B)*** Interaction surface between two monomers that form the trimeric ring. The forces that stabilise the trimeric ring include salt bridges as well as hydrophobic interactions between the N-terminal of a single monomer to the C-terminal of a nearby monomer in a continuous manner.

**Fig 4:**
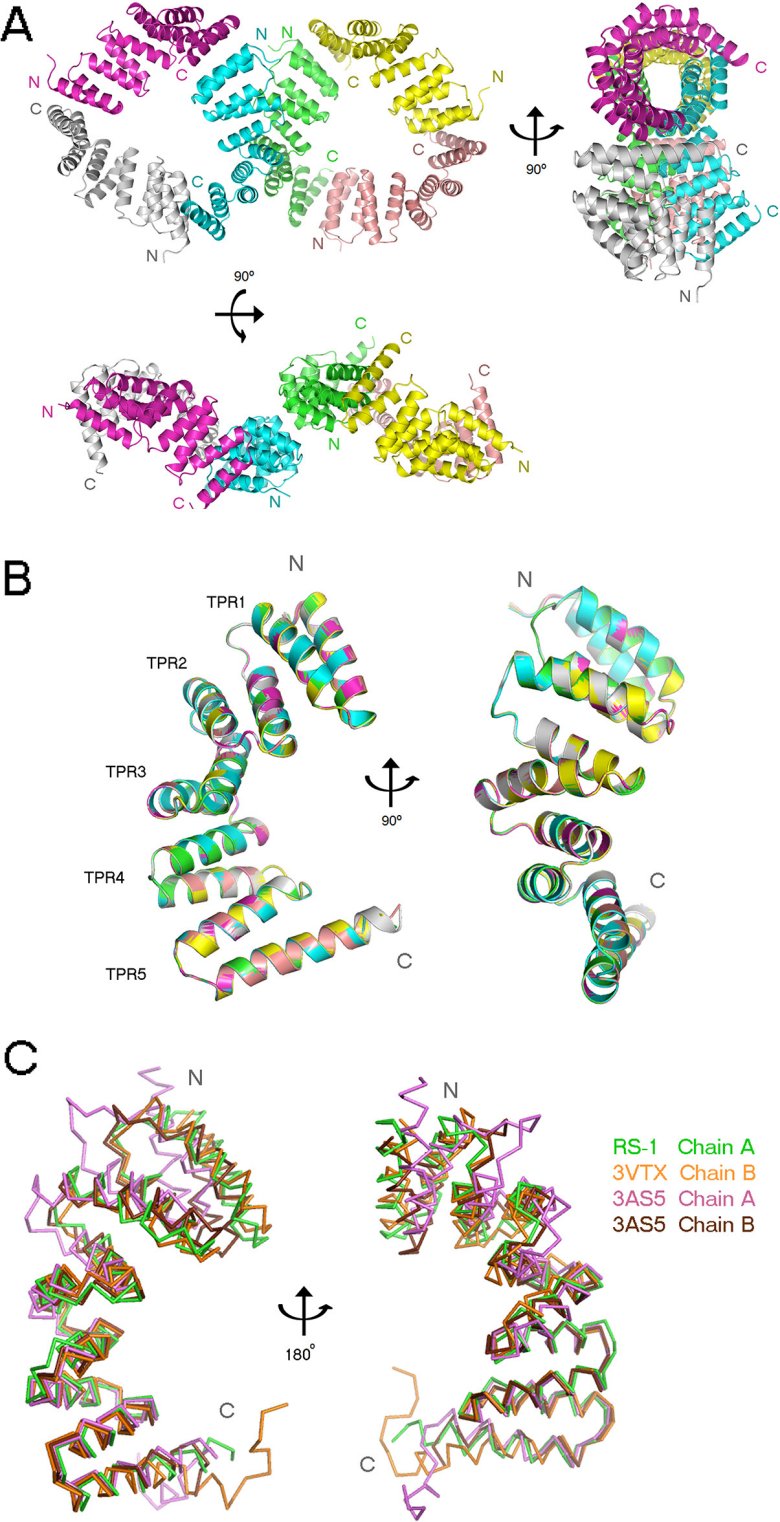
ArsTM crystal packing, asymmetric unit composition and overall structure. ***(A)*** ArsTM crystal packing and asymmetric unit composition. The molecules are shown in three rotation-related views. ***(B)*** Overlay of all six ArsTM monomers reveals the high degree of structural similarity. The representative ArsTM monomer contains five sequential TPR motifs. The molecule is shown in two views, related by a 90° rotation. ***(C)*** An overlay of representative monomers from ArsTM (green), MamAΔ41_Mbav_ (PDB ID: 3VTX, orange) and *Magnetospirillum* species MamAΔ41_AMB-1_ (PDB ID: 3AS5 chain A and B in light pink and brown, respectively) related by a 180° rotation. A high structural similarity of MamAΔ41 between the species can be observed, apart from the helical conformation of the identical His-tag linker sequence remaining after thrombin proteolysis (H11: ELALVPR) seen in the 3AS5 chain B and 3VTX. In addition, a light flexibility is observed at the NTD of the monomers. All images were produced by PyMOL.

ArsTM monomers assemble to form a trimeric ring, each enclosing a ~15 Å diameter inner void (Fig 3A); the crystal asymmetric unit includes six ArsTM monomers arranged as two trimeric rings (Fig 4A). The employed forces that allow the stabilisation of each trimeric ring include salt bridges as well as hydrophobic interactions between the N-terminal of a single monomer and the C-terminal of a nearby monomer in a continuous manner, resulting in a ring with surface properties akin to a Möbius strip. These ring-stabilising salt bridges include a double salt bridge between Glu61 and Tyr65 from a single monomer to Gln183 from the nearby monomer, as well as a single salt bridge between Arg56 from a single monomer to Glu209 from the nearby monomer. The network of hydrophobic interactions includes Lys42, Leu45, Tyr46, Ile49, Arg52, Ser53, Arg64 and Glu68 from the N-terminal of a single monomer to Phe187, Val190, Ala198, Ala199, Phe202, Val205 and Met206 to the C-terminal of the nearby monomer (Fig 3B). As such, ArsTM presents a distinctly atypical binding surface since, as mentioned, the protein-protein interactions involving MamA typically bind via the concave or convex surfaces. Overall, the trimeric ring contains three of these identical, N-to-C-terminal interaction surfaces. Unexpectedly, but not altogether unsurprisingly, the triple mutated residues (E140A, K141A and E143A), along the ‘backbone’ of the monomers, were found to be at the centre of a crystal contact (S3 Fig). The tight packing of ArsTM within the crystal could not occur with the original residues, which contained long and charged side chains and hence, could not be crystallised. This interaction surface includes two symmetric backbone polar contacts between two monomers (Phe111 to Ala140), where each monomer originates from a different ring. In addition, the interaction surface is stabilised through double hydrophobic interactions between Pro142 to His 110 (S3 Fig).

### ArsTM overall structure

The overall structure of the ArsTM monomer contains 10 anti-parallel α-helices, folded as five TPR helix-turn-helix motifs, namely TPR1 (H1 and H2), TPR2 (H3 and H4), TPR3 (H5 and H6), TPR4 (H7 and H8) and TPR5 (H9 and H10), similar to previously determined MamA structures (Fig 4B). These TPR motifs give rise to a structure displaying concave and convex surfaces. All six ArsTM monomers adopted the same overall fold, as the average root mean square deviation (RMSD) between the Cα of the monomers ranges between 0.08–0.1 Å (Fig 4B). Additionally, we tested for the secondary sturcture of the protein in solution using circular dichroism (see below, *NTD stabilisation and the functionality of the conserved salt bridge*). Overall, all MamA variants are folded as alpha helices, as predited for a TPR-containing protein. Our results show highly similar structures for ArsTM, wild type MamAΔ41 RS-1 and MamA from Mbav, indicating that our mutations had no alteration effect on the helices of MamAΔ41 RS-1. Whilst MamA from both AMB-1 and MSR-1 display slight deviation from the other three MamA proteins, they all still remain highly similar. Overlapping ArsTM on MamA structures from *Magnetospirillum magneticum* AMB-1, *Magnetospirillum gryphiswaldense* MSR-1 and Mbav also yielded low RMSD values ranging between 1.26–1.70 Å (Fig 4C and S2 Table) [11]. These low RMSD values together with the sequence conservation indicate that the MamAΔ41 structure is conserved across phyla, which runs counter to the hypothesis of HGT.

Like MamAΔ41 from *Magnetospirillum* and Mbav species, ArsTM also displays the typical electrostatic charge distribution, with its concave surface potential being mainly positive. However, the convex surface potential of ArsTM contains a mixture of positive and negative patches and thus is more similar to MamAΔ41Mbav convex surface, while in *Magnetospirillum* species this region is mainly negative (S4 Fig).

Previously determined MamAΔ41 structures from *Magnetospirillum* and Mbav species displayed a unique separation into two distinct domains [11]. The hinge region that separates between the N-terminal domain (NTD), which includes TPR motifs 1–2, and the C-terminal domain (CTD), which includes TPR motifs 3–5, is located at the loop connecting TPR2 to TPR3. This flexible hinge region allows a radial movement of the NTD in reference to the CTD upon binding of a putative ligand at the concave surface [11]. In ArsTM we could not detect such domain movement, as the conformations of all six monomers are similar. However, by overlapping ArsTM monomers onto the available MamAΔ41 structures that did display such NTD movement, we could see that the curvature of ArsTM is more similar to the putative ligand binding conformation (i.e. MamAΔ41_AMB-1_ PDB code: 3AS5 chain B, and MamAΔ41_Mbav_ PDB code: 3VTX) (Fig 4C and S1 Table). Such curvature of ArsTM, though no putative ligand was observed at the crystal structure, could have been more favourable due to the trimeric rings’ local packing as well as other crystal contacts.

The five-TPR set-up and its associated hinge region further separate MamA from other TPR-containing proteins; other TPR proteins generally possess either far fewer repeats (approximately three), to allow for domain separation, or far greater, resulting in a continuous superhelical structure, free of the kinked hinge region found in MamA. The high degree of sequence variation surrounding the structurally conserved hinge region indicates that there was sufficient time for this evolution to occur, counter to the structural- and sequence-homogeneity that would occur via HGT. That all thus-far described MamA proteins from various species possess these structural motifs resulting in the same, overall structure, unique among TPR proteins, further indicates for a single origin that has undergone evolutionary sequence changes over time but retained both its overall structural elements and its function within the various species of MTB.

Multiple sequence alignment between ArsTM and MamA from other species shows a distinct lack of conservation around the connecting loops between the helices, including the hinge region described above (S2 Fig). These regions are similar in the *Magnetospirillum* spp. MTB but there is significant variation for MamA from the deeply-branching RS-1 and “*Ca*. M. bavaricum”; we hypothesise this to be due to the loops not participating in any protein-protein interactions and thus there is no need for these to remain conserved.

### NTD stabilisation and the functionality of the conserved salt bridge

**Fig 5:**
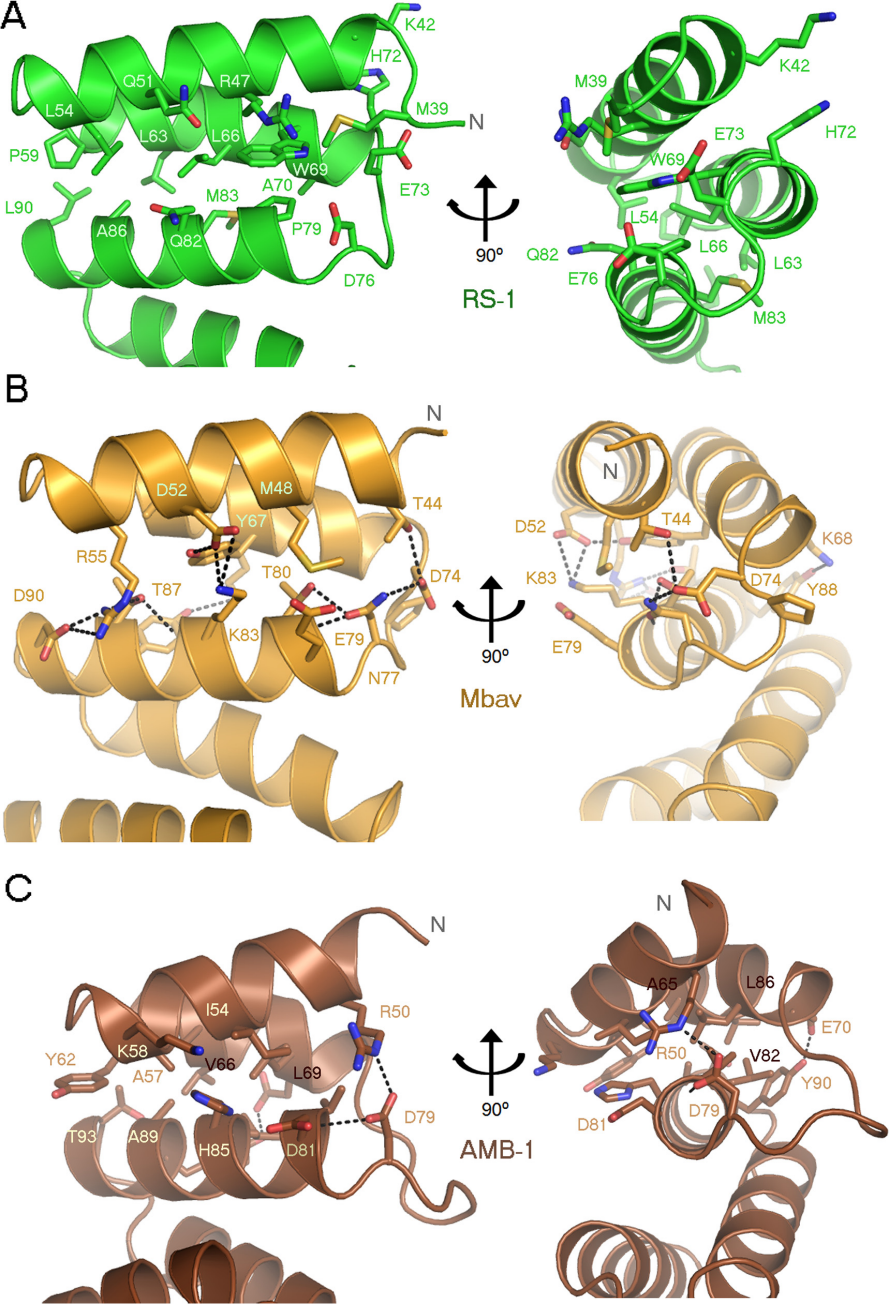
NTD stabilisation of MamAΔ41 from different species. ***(A)*** Detailed representation of the interactions stabilising the ArsTM NTD. The ArsTM NTD is stabilised by a diverse network of hydrophobic interactions. ***(B)*** Detailed representation of the interactions stabilising the MamAΔ41_Mbav_ NTD. The MamAΔ41_Mbav_ NTD is stabilised by a diverse network of hydrogen bonds and a single non-conserved salt bridge. ***(C)*** Detailed representation of the interactions stabilising the MamAΔ41_AMB-1_ NTD. The MamAΔ41_AMB-1_ NTD is stabilised through numerous hydrophobic interactions, a few hydrogen bonds and a single conserved salt bridge. Both NTD domains are shown in two views, related by a 90° rotation.

MamA proteins from different species display a high degree of sequence and structural similarity (S2 Fig) [11,19]. Most of the fully conserved residues are related to the TPR motif consensus sequence apart from Asp159. Previous work on *Magnetospirillum* species revealed that mutating Asp159 had no effect on the overall structure of MamAΔ41, MamA complex formation or the *in vivo* sub-cellular localisation to the magnetosome chain [19]. Nevertheless, it is possible that Asp159 mediates other cellular protein-protein interactions with MamA. In addition to Asp159, two other MamA residues, Arg50 and Asp79, were considered to be highly conserved in all available species prior to the release of Mbav and BW-1 (a magnetotactic *Deltaproteobacteria*) sequences. The determined structures of MamA from *Magnetospirillum* species revealed that Arg50 and Asp79 form a salt bridge that stabilises the NTD by connecting the TPR1 and TPR2 motifs. Whilst these salt bridge-forming residues are conserved in ArsTM (Arg47 and Asp76), the salt bridge itself is not since the distance between these two residues is too great (Fig 5A). Furthermore, due to steric interference by the large Trp69, clearly shown in the structure, we could find no rotameric combinations for Arg47 and Asp76 capable of forming the salt bridge around Trp69. Trp69 occupies a large volume and is part of an extensive hydrophobic surface that stabilises the NTD packing. We presume that, due to the large volume of the Trp69 side chain, motifs TPR1 and TPR2 cannot be packed as tightly as their homologous motifs from *Magnetospirillum* species and MamAΔ41_Mbav_ (Fig 5B & 5C).

In other species, it has been found that disruption of this salt bridge altered the orientation of the NTD in relation to the CTD, eliminated the ability of MamA to homo-oligomerise and caused *in vivo* protein mislocalisation [19]. Despite having such a significant structural role, these conserved salt bridge-forming residues were altered in MamA sequences from Mbav and BW-1. An explanation for the remaining functionality of MamAΔ41Mbav NTD, although losing its conserved salt bridge, was previously suggested; namely that broad electrostatic interactions between the first two TPR motifs can compensate for the loss of this conserved salt bridge [11]. As such, evolution may have yielded a solution to the loss of the salt bridge whilst retaining the overall structure.

Despite Trp69 steric interference, we believe that the NTD domain of ArsTM remains stable due to the hydrophobic pi-pi stacking formed between Trp69 and Arg47. The overall interactions which stabilise the NTD of ArsTM are solely hydrophobic interactions and involve residues: Met39, Lys42, Arg47, Gln51, Leu54, Pro59, Leu63, Leu66, Trp69, Ala70, His72, Pro79, Gln83, Met83, Ala86 and Leu90 (Fig 5A). Such stabilisation through hydrophobic interactions is considered to be weaker than stabilisation through polar interaction and salt bridges, as observed in the NTDs of MamAΔ41 from *Magnetospirillum* species and MamAΔ41_Mbav_. MamAΔ41_Mbav_ presents the tightest and most stable interaction surface, stabilised by an extensive hydrogen bond network at the surface of TPR1 and TPR2, along with a salt bridge, and around the TPR2-3 connecting loop. A more moderate and intermediate stabilisation pattern can be seen in MamAΔ41 from *Magnetospirillum* species, which presents a mixture of salt bridge, polar and hydrophobic interactions. ArsTM, on the other hand, lacks the stabilising network of hydrogen bonds but displays tight packing between TPR1 and TPR2, achieved mainly by a hydrophobic interaction network (Fig 5A).

**Fig 6:**
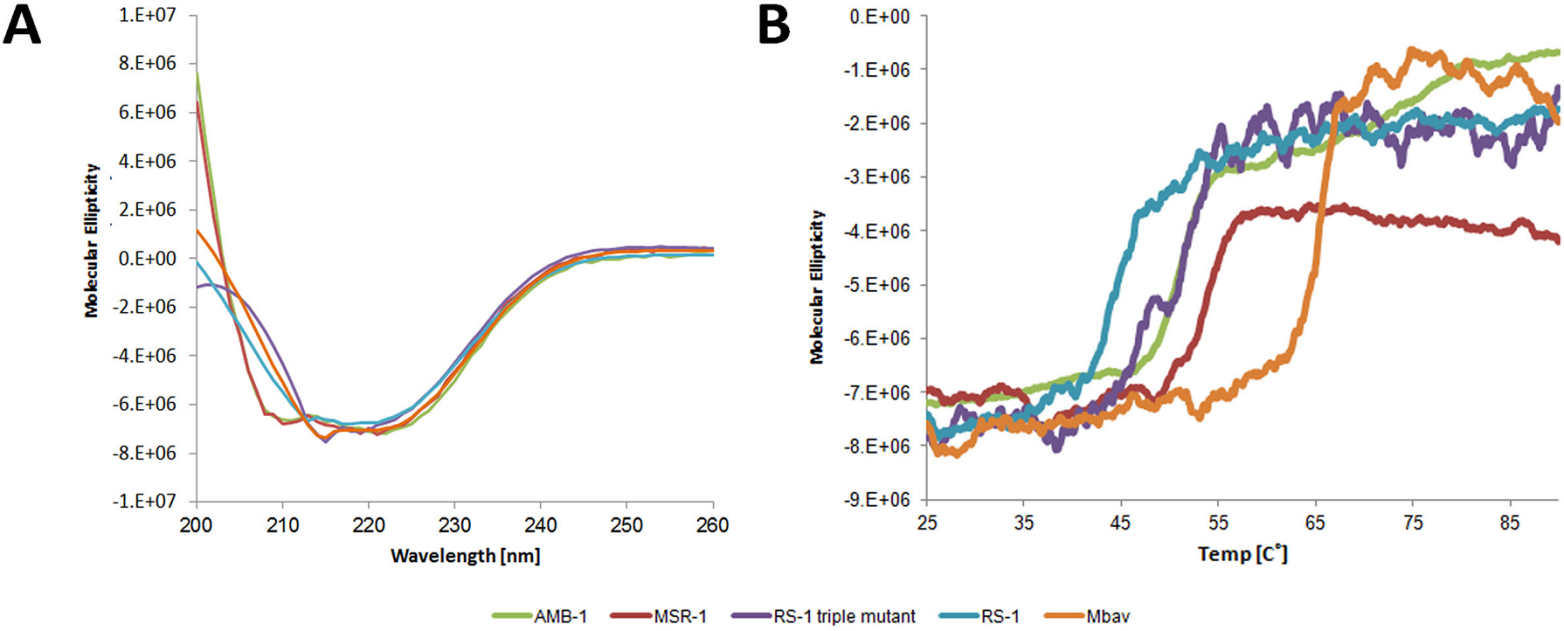
Circular dichroism measurements of ArsTM (purple) and MamAΔ41 proteins from RS-1 (Blue) Mbav (orange), AMB-1 (green) and MSR-1 (red). (A) Circular dichroism spectra. (B) Circular dichroism melting curve measurements at 222 nm. Wild type MamAΔ41_RS-1_ presents the lowest thermostability, with a melting temperature of ~40°C, while the triple mutated MamAΔ41_RS-1_ (ArsTM) exhibits a slightly increased thermostability with a melting temperature of ~ 51°C. MamAΔ41_AMB-1_, MamAΔ41_MSR-1_ and MamAΔ41_Mbav_ present melting temperatures of ~51, 53 and 65°C, respectively.

The described variations between stabilisation patterns of the NTD among MamA proteins from different species can be linked to the overall thermostability seen in circular dichroism spectrometry. Melting temperature measurements revealed that MamAΔ41_RS-1_ presents a reduced thermostability, with a melting temperature of 45°C, while MamAΔ41_AMB-1_, MamAΔ41_MSR-1_ and MamAΔ41_Mbav_ present melting temperatures of 51, 53 and 65°C, respectively (Fig 6). The most thermostable protein is MamAΔ41_Mbav_, which employs only polar interactions to stabilise its NTD, whereas the least thermostable protein is MamAΔ41_RS-1_, which employs only hydrophobic interactions. In between this thermostability range we can find MamAΔ41 from *Magnetospirillum* species, which employ both types of interactions. We could also see that subjecting MamAΔ41_RS-1_ to the triple surface entropy reduction mutations increased its thermostability by 6°C, by which it reached the same temperature as MamAΔ41_AMB-1_. In order to obtain coherent and comparable results, we examined the octameric forms for both MamAΔ41_RS-1_ and ArsTM during the circular dichroism spectrometry measurements. Accordingly, we believe that such slightly increased thermostability is due to the increased structural stability between ArsTM monomers when assembled as oligomers in solution (S1 Fig).

## Conclusion

Our studies reveal that the structure of MamA has remained remarkably conserved throughout evolution with minimal change across different taxa. In particular, there is a high degree of structural conservation for the hinge region, CTD and NTD, indicative for a similar–yet unknown–function for MamA in all MTB, despite sequence variations. As such, MamA may be the most structurally conserved magnetosome-associated protein. This structural conservation is not limited to a particular motif but runs throughout the entire structure, from the five-TPR superhelical structure through to the hinge region. Our data enabled us to create evolutionary trees for MamA based on the amino acid sequences as well as on the 16S rRNA. A comprehensive approach to these data, together with the sequence variation and solutions to maintain the structural effects of the salt bridge, indicates that magnetotaxis likely did not spread via horizontal gene transfer and instead has a significantly earlier, primordial origin.

## Materials and Methods

### Protein Expression and Purification

Both full length and truncated MamA were amplified by whole-cell polymerase chain reaction (PCR) from *Desulfovibrio magneticus* RS-1 (kindly provided by Prof. Arash Komeili, Berkley, California, USA). Amplified products contained an inserted N-terminal NcoI restriction site followed by a glycine codon (GGA) in order to maintain reading frame and a C-terminal SacI restriction site eliminating the gene’s normal stop codon. Amplified products were recovered, digested and ligated into pET52(b) expression vector (Novagen) containing a C-terminal histidine_10_ tag. An additional, seemingly silent, spontaneous mutation was discovered during gene sequencing at residue M124I. Transformed *E*. *coli* Rosetta strain cells were cultivated in auto-induction medium [27] containing ampicillin and chloramphenicol (100 mg ml^-1^ and 30 mg ml^-1^ respectively). Cultivated cells were kept at 310 K for 6 hours and then at 300 K for an additional 60 hours. Cells were collected by centrifugation at 7438 g for 8 min at 277 K. Protein purification steps were based on previously published purification protocols for MamAΔ41 from *Magnetospirillum magneticum* AMB-1 and *Magnetospirillum gryphiswaldense* MSR-1 [11,19–21]. In short, MamAΔ41-expressing cells were resuspended in buffer A (50 mM HEPES pH 7.5, 0.5 M NaCl, 20 mM imidazole and 5 mM βME) with the addition of DNase I (10 mg ml^-1^) and a cocktail of protease inhibitors (100 μM phenylmethylsulfonyl fluoride (PMSF), 1.2 μg ml^-1^ leupeptin and 1 μM pepstatin A). Cell disruption was performed by two cycles of French press pressure cell at 172 MPa and the soluble protein fraction was differentiated by centrifugation at 45,000 g for 90 min at 277 K. The soluble fraction was applied onto a homemade Ni-NTA column (HisPur Ni-NTA resin, Thermo scientific) pre-equilibrated against buffer A. Subsequent to protein binding, the column was step-washed by 50 ml of buffer B (20 mM HEPES pH 7.5, 1 M NaCl, 40 mM imidazole and 5 mM βME), 50 ml of buffer C (20 mM HEPES pH 7.5, 0.5 M NaCl, 40 mM imidazole and 5 mM βME) and eluted by buffer D (20 mM HEPES pH 7.5, 0.5 M NaCl, 0.5 M imidazole and 5 mM βME). The eluted protein was dialysed against buffer E (10 mM HEPES pH 7.5, 0.5 M NaCl and 5 mM βME) for 12 hours at 277 K followed by concentration using an Amicon Ultracel (3 kDa cutoff, Millipore), to a final volume of 2.5 ml. Concentrated protein was applied onto a size-exclusion column (Superdex 200, GE Healthcare Biosciences) pre-equilibrated with buffer E. Relevant protein peaks were merged, concentrated and flash frozen in liquid nitrogen and stored at 193 K for crystallisation trails. The ArsTM peaks that corresponded to monomeric and octameric protein forms were concentrated separately to 36 mg ml^-1^ and 28 mg ml^-1^, respectively.

### Site-directed mutagenesis by PCR

The triple mutant (ArsTM) was generated using QuikChange site-directed mutagenesis (Stratagene). Coding and anti-sense primers containing a single mutagenic site were used for PCR amplification.

### Crystallisation and diffraction collection

Crystallisation trials of ArsTM were conducted using the sitting-drop vapour-diffusion method. Initial screenings were performed using commercial screening kits obtained from Molecular Dimensions (Structure screen) and Hampton Research (Index screen) at 286 K and 277 K. Each drop contained a mixture of 0.2 μl of sampled reservoir and 0.2 μl of monomeric protein solution (28 mg ml^-1^). Crystal harvesting was performed from optimised conditions of 0.1 M Tris pH 8.1, 0.75 M sodium potassium tartrate and 0.5% polyethylene glycol monomethyl ether 5000. The crystals were then soaked for 5 seconds in a drop containing the same conditions, as well as 40% glycerol, serving as a cryo-protectant agent. Glycerol-protected crystals were then flash-cooled in liquid nitrogen. Diffraction dataset was collected on beamline ID14-4 at the European Synchrotron Radiation Facility (ESRF), Grenoble, France. Data was measured at the 0.939 Å wavelength for 230 images at an oscillation range of 0.65°, an exposure time of 0.375 sec per image and a crystal-to-detector distance of 422.81 nm. Data were reduced and scaled using the HKL2000 program suite [28]. Phase acquisitions and structure determinations were performed using *BALBES*: *a molecular replacement pipeline* [29] followed by auto-build cycles using ARP/wARP [30]. The final model was built by Coot and refined in REFMAC5 [31]. For Rfree calculation, 5% of the data were excluded from both data sets. Structural figures were prepared using PyMOL [32] and Coot.

### Least-squares overlaps

R.M.S. calculations were performed with SwissPDB viewer [33] using the domain alternate fit on Cα to align structures on the basis of the conserved domain and to define the conformational changes of the structural homologues.

### Electrostatic potential calculation

Calculations were performed in PyMOL using the adaptive Poisson-Boltzman solver (APBS) plug-in [34].

### Circular dichroism (CD) analysis

CD measurements were conducted with a J750 Spectropolarimeter (Jasco, Mary's Court, Easton, USA) equipped with a Pelletier device. MamA protein samples were pre-diluted to 0.15 mg ml^-1^ in buffer containing 150 mM NaCl, 10 mM Tris-HCl, pH 8, and measured with a 0.1 cm optical path suprasil quartz cuvette (Hellma, Müllheim, Germany). Thermal denaturation experiments of both samples were conducted by monitoring the dichroic absorption at 222 nm as a function of increased temperature varying from 25 to 90°C at a heating rate of 2.0°C·min^-1^. Thermodynamic parameters associated with the temperature-induced denaturation of the protein were obtained by nonlinear, least-squares analysis of the temperature dependence of the CD spectrum.

### Multiple sequence alignment and generation of phylogenetic trees

Initial multiple alignments were generated using the CLUSTAL W multiple alignment accessory application in the BioEdit sequence alignment editor [35]. Alignment was visualized using Jalview package [36]. Evolutionary trees were obtained using the MEGA 6 package [37] applying the Maximum Likelihood algorithm [38]. Bootstrap values were calculated with 100 replicates.

### MamA protein sequences

All MamA sequences were obtained from GenBank. All accession numbers are shown in the phylogenetic tree in Fig 1.

### Coordinates

Structure and structure factors have been submitted to the Protein Data Bank (accession codes: 4XI0).

## Supporting Information

S1 Fig Oligomeric state of purified MamAΔ41 according to size exclusion (Superdex 200) chromatograms from different species. Elution profiles of MamAΔ41 triple mutant from *Desulfovibrio magneticus* (RS-1) and wild type MamAΔ41 from *Desulfovibrio magneticus* (RS-1), *M*. *magneticum* (AMB-1), *M*. *gryphiswaldense* (MSR-1) and *Candidatus Magnetobacterium bavaricum* (Mbav) colored in light blue, green, red, orange and blue, respectively. Wild type MamAΔ41 from RS-1 eluted in a volume corresponds to octamer (~192 kDa) whereas the triple mutated MamAΔ41 eluted in three separate peaks that correspond to a 13-monomer oligomer (~312 kDa), octamer (~192 kDa) and a monomer (~ 24 kDa). Both MamAΔ41 from AMB-1 and Mbav eluted at a volume corresponding to the monomer (20–22 kDa). MamAΔ41 from MSR-1 eluted at a volume typical of the trimer (~60 kDa). Dashed green line represents the elution profile of protein markers: Ferrritin (~440 kDa), Ovalbumin (~43 kDa), Carbonic Anhydrase (~29 kDa), Ribonuclease (~14 kDa).
(DOCX)

S2 Fig Multiple sequence alignment of all 21 complete available MamA sequences from cultivated and uncultivated magnetotactic bacteria for which the 16S rRNA gene sequence is known. The MTB from *Alphaproteobacteria* class used in the analyses are: *Magnetospirillum magnetotacticum* (strain MS-1), *Ms*. *magneticum* (AMB-1), *Ms*. *gryphiswaldense* (MSR-1), strain SO-1, strain LM-1, *Magnetovibrio blakemorei* (MV-1), *Magnetospira* sp. QH-2, strain MO-1, *Magnetofaba australis* (IT-1) and *Magnetococcus marinus* (MC-1). Strain SS-5 from the *Gammaproteobacteria* class is also used. From the *Deltaproteobacteria* class MTB used include the magnetotactic multicellular prokaryotes *Ca*. Magnetoglobus multicellularis (MMP) and strain HK-1, *Ca*. Desulfamplus magnetomortis (BW-1), *Desulfovibrio magneticus* (RS-1 and FH-1), and strain ML-1. *Ca*. Magnetobacterium bavaricum (Mbav) and strain MYR-1 of the *Nitrospirae* phylum was also used. Red numbers at the bottom denote residue numbers specific for ArsTM.
(DOCX)

S3 Fig Crystal contacts between two ArsTM monomers. The triple mutated residues (E140A, K141A and E143A, highlighted as red spheres it the top view) are found in the centers of these interaction surfaces.
(DOCX)

S4 Fig Surface charge comparison of MamAΔ41 structures. Surface charge comparison of MamAΔ41 structures, with blue and red colours representing regions of positive and negative electrostatic potential, respectively. The molecule is shown in three views, namely the concave surface, a side view and the convex surface, related by 90° rotations. The surface charge representation of ArsTM and MamAΔ41Mbav display a concave surface that is mainly positive and a convex surface that contains both positive and negative patches. The surface charge representation of MamAΔ41AMB-1 displays a concave surface that is extremely positive and a mainly negative convex surface. All electrostatic surfaces representations were produced with the APBS plug-in of PyMOL under the same contour levels.
(DOCX)

S1 Table MamAΔ41 structure: Ramachandran statistics and missing residues. (DOCX)

S2 Table RMSD values of MamAΔ41 monomers from alternative species compared against ArsTM. (DOCX)
