# Supplementary material for: MamA as a Model Protein for Structure-Based Insight into the Evolutionary Origins of Magnetotactic Bacteria
Source: PLoS One. 2015 Jun 26;10(6):e0130394. doi: 10.1371/journal.pone.0130394 (PMC4482739; doi:10.1371/journal.pone.0130394)
Supplement: S1 Fig — Elution profiles of MamAΔ41 triple mutant from Desulfovibrio magneticus (RS-1) and wild type MamAΔ41 from Desulfovibrio magneticus (RS-1), M. magneticum (AMB-1), M. gryphiswaldense (MSR-1) and Candidatus Magnetobacterium bavaricum (Mbav) colored in light blue, green, red, orange and blue, respectively. Wild type MamAΔ41 from RS-1 eluted in a volume corresponds to octamer (~192 kDa) whereas the triple mutated MamAΔ41 eluted in three separate peaks that correspond to a 13-monomer oligomer (~312 kDa), octamer (~192 kDa) and a monomer (~ 24 kDa). Both MamAΔ41 from AMB-1 and Mbav eluted at a volume corresponding to the monomer (20–22 kDa). MamAΔ41 from MSR-1 eluted at a volume typical of the trimer (~60 kDa). Dashed green line represents the elution profile of protein markers: Ferrritin (~440 kDa), Ovalbumin (~43 kDa), Carbonic Anhydrase (~29 kDa), Ribonuclease (~14 kDa). (DOCX) [file pone.0130394.s001.docx]

**Fig. S1 – Oligomeric state of purified MamAΔ41 according to size exclusion (Superdex 200) chromatograms from different species.** Elution profiles of MamAΔ41 triple mutant from *Desulfovibrio magneticus* (RS-1) and wild type MamAΔ41 from *Desulfovibrio magneticus* (RS-1), *M. magneticum* (AMB-1), *M. gryphiswaldense* (MSR-1) and *Candidatus Magnetobacterium bavaricum* (Mbav) colored in light blue, green, red, orange and blue, respectively. Wild type MamAΔ41 from RS-1 eluted in a volume corresponds to octamer (~192 kDa) whereas the triple mutated MamAΔ41 eluted in three separate peaks that correspond to a 13-monomer oligomer (~312 kDa), octamer (~192 kDa) and a monomer (~ 24 kDa). Both MamAΔ41 from AMB-1 and Mbav eluted at a volume corresponding to the monomer (20-22 kDa). MamAΔ41 from MSR-1 eluted at a volume typical of the trimer (~60 kDa). Dashed green line represents the elution profile of protein markers: Ferrritin (~440 kDa), Ovalbumin (~43 kDa), Carbonic Anhydrase (~29 kDa), Ribonuclease (~14 kDa).
