# Supplementary material for: MamA as a Model Protein for Structure-Based Insight into the Evolutionary Origins of Magnetotactic Bacteria
Source: PLoS One. 2015 Jun 26;10(6):e0130394. doi: 10.1371/journal.pone.0130394 (PMC4482739; doi:10.1371/journal.pone.0130394)
Supplement: S2 Fig — The MTB from Alphaproteobacteria class used in the analyses are: Magnetospirillum magnetotacticum (strain MS-1), Ms. magneticum (AMB-1), Ms. gryphiswaldense (MSR-1), strain SO-1, strain LM-1, Magnetovibrio blakemorei (MV-1), Magnetospira sp. QH-2, strain MO-1, Magnetofaba australis (IT-1) and Magnetococcus marinus (MC-1). Strain SS-5 from the Gammaproteobacteria class is also used. From the Deltaproteobacteria class MTB used include the magnetotactic multicellular prokaryotes Ca. Magnetoglobus multicellularis (MMP) and strain HK-1, Ca. Desulfamplus magnetomortis (BW-1), Desulfovibrio magneticus (RS-1 and FH-1), and strain ML-1. Ca. Magnetobacterium bavaricum (Mbav) and strain MYR-1 of the Nitrospirae phylum was also used. Red numbers at the bottom denote residue numbers specific for ArsTM. (DOCX) [file pone.0130394.s002.docx]

**Fig. S2** – Multiple sequence alignment of all 21 complete available MamA sequences from cultivated and uncultivated magnetotactic bacteria for which the 16S rRNA gene sequence is known. The MTB from *Alphaproteobacteria* class used in the analyses are: *Magnetospirillum magnetotacticum* (strain MS-1), *Ms. magneticum* (AMB-1), *Ms. gryphiswaldense* (MSR-1), strain SO-1, strain LM-1, *Magnetovibrio blakemorei* (MV-1), *Magnetospira* sp. QH-2, strain MO-1, *Magnetofaba australis* (IT-1) and *Magnetococcus marinus* (MC-1). Strain SS-5 from the *Gammaproteobacteria* class is also used. From the *Deltaproteobacteria* class MTB used include the magnetotactic multicellular prokaryotes *Ca*. Magnetoglobus multicellularis (MMP) and strain HK-1, *Ca*. Desulfamplus magnetomortis (BW-1), *Desulfovibrio magneticus* (RS-1 and FH-1), and strain ML-1. *Ca*. Magnetobacterium bavaricum (Mbav) and strain MYR-1 of the *Nitrospirae* phylum was also used. Red numbers at the bottom denote residue numbers specific for ArsTM.
