# Supplementary material for: MamA as a Model Protein for Structure-Based Insight into the Evolutionary Origins of Magnetotactic Bacteria
Source: PLoS One. 2015 Jun 26;10(6):e0130394. doi: 10.1371/journal.pone.0130394 (PMC4482739; doi:10.1371/journal.pone.0130394)
Supplement: S3 Fig — The triple mutated residues (E140A, K141A and E143A, highlighted as red spheres it the top view) are found in the centers of these interaction surfaces. (DOCX) [file pone.0130394.s003.docx]

**Fig. S3 – Crystal contacts between two ArsTM monomers.** The triple mutated residues (E140A, K141A and E143A, highlighted as red spheres it the top view) are found in the centers of these interaction surfaces.
