# Supplementary material for: MamA as a Model Protein for Structure-Based Insight into the Evolutionary Origins of Magnetotactic Bacteria
Source: PLoS One. 2015 Jun 26;10(6):e0130394. doi: 10.1371/journal.pone.0130394 (PMC4482739; doi:10.1371/journal.pone.0130394)
Supplement: S4 Fig — Surface charge comparison of MamAΔ41 structures, with blue and red colours representing regions of positive and negative electrostatic potential, respectively. The molecule is shown in three views, namely the concave surface, a side view and the convex surface, related by 90° rotations. The surface charge representation of ArsTM and MamAΔ41Mbav display a concave surface that is mainly positive and a convex surface that contains both positive and negative patches. The surface charge representation of MamAΔ41AMB-1 displays a concave surface that is extremely positive and a mainly negative convex surface. All electrostatic surfaces representations were produced with the APBS plug-in of PyMOL under the same contour levels. (DOCX) [file pone.0130394.s004.docx]

**Fig. S4 – Surface charge comparison of MamAΔ41 structures.** Surface charge comparison of MamAΔ41 structures, with blue and red colours representing regions of positive and negative electrostatic potential, respectively. The molecule is shown in three views, namely the concave surface, a side view and the convex surface, related by 90⁰ rotations. The surface charge representation of ArsTM and MamAΔ41Mbav display a concave surface that is mainly positive and a convex surface that contains both positive and negative patches. The surface charge representation of MamAΔ41AMB-1 displays a concave surface that is extremely positive and a mainly negative convex surface. All electrostatic surfaces representations were produced with the APBS plug-in of PyMOL under the same contour levels.
