# Supplementary material for: MamA as a Model Protein for Structure-Based Insight into the Evolutionary Origins of Magnetotactic Bacteria
Source: PLoS One. 2015 Jun 26;10(6):e0130394. doi: 10.1371/journal.pone.0130394 (PMC4482739; doi:10.1371/journal.pone.0130394)
Supplement: S1 Table — (DOCX) [file pone.0130394.s005.docx]

**Table S1** – MamAΔ41 structure: Ramachandran statistics and missing residues.

| **aberration** | MamAΔ41-TM_RS-1_ |
| --- | --- |
| **Space group** | I_4_ |
| **Ramachandran statistics^Φ^** | A: 1000 (95.60%) |
|  | P :45 (4.30%) |
|  | D: 1 (0.10%) |
| **Missing residues including the Thrombin proteolysis site and 10X His tag** | A 215-237 |
|  | B 213-237 |
|  | C 213-237 |
|  | D 214-237 |
|  | E 215-237 |
|  | F 217-237 |
|  |  |
| (Φ) A- fully allowed region, P- partially allowed region and D- disallowed region | |
