# Supplementary material for: MamA as a Model Protein for Structure-Based Insight into the Evolutionary Origins of Magnetotactic Bacteria
Source: PLoS One. 2015 Jun 26;10(6):e0130394. doi: 10.1371/journal.pone.0130394 (PMC4482739; doi:10.1371/journal.pone.0130394)
Supplement: S2 Table — (DOCX) [file pone.0130394.s006.docx]

**Table S2** - RMSD values of MamAΔ41 monomers from alternative species compared against ArsTM.

| **First monomer** | | **Second monomer** | | **Cα RMSD [Å]** |
| --- | --- | --- | --- | --- |
| RS-1 | ArsTM _chain A | RS-1 | ArsTM _chain A | 0.00 |
|  | ArsTM _chain A |  | ArsTM _chain B | 0.08 |
|  | ArsTM _chain A |  | ArsTM _chain C | 0.09 |
|  | ArsTM _chain A |  | ArsTM _chain D | 0.10 |
|  | ArsTM _chain A |  | ArsTM _chain E | 0.10 |
|  | ArsTM _chain A |  | ArsTM _chain F | 0.09 |
|  | ArsTM _chain A | Mbav | 3VTX_chain A | 1.38 |
|  | ArsTM _chain A |  | 3VTY_chain A | 1.48 |
|  | ArsTM _chain A | AMB-1 | 3AS5_chain A | 1.46 |
|  | ArsTM _chain A |  | 3AS5_chain B | 1.29 |
|  | ArsTM _chain A | MSR-1 | 3AS8 | 1.70 |
