# Supplementary figures and images for: Correction: MamA as a Model Protein for Structure-Based Insight into the Evolutionary Origins of Magnetotactic Bacteria
Source: PLoS One. 2015 Jul 15;10(7):e0133556. doi: 10.1371/journal.pone.0133556 (PMC4503739; doi:10.1371/journal.pone.0133556)

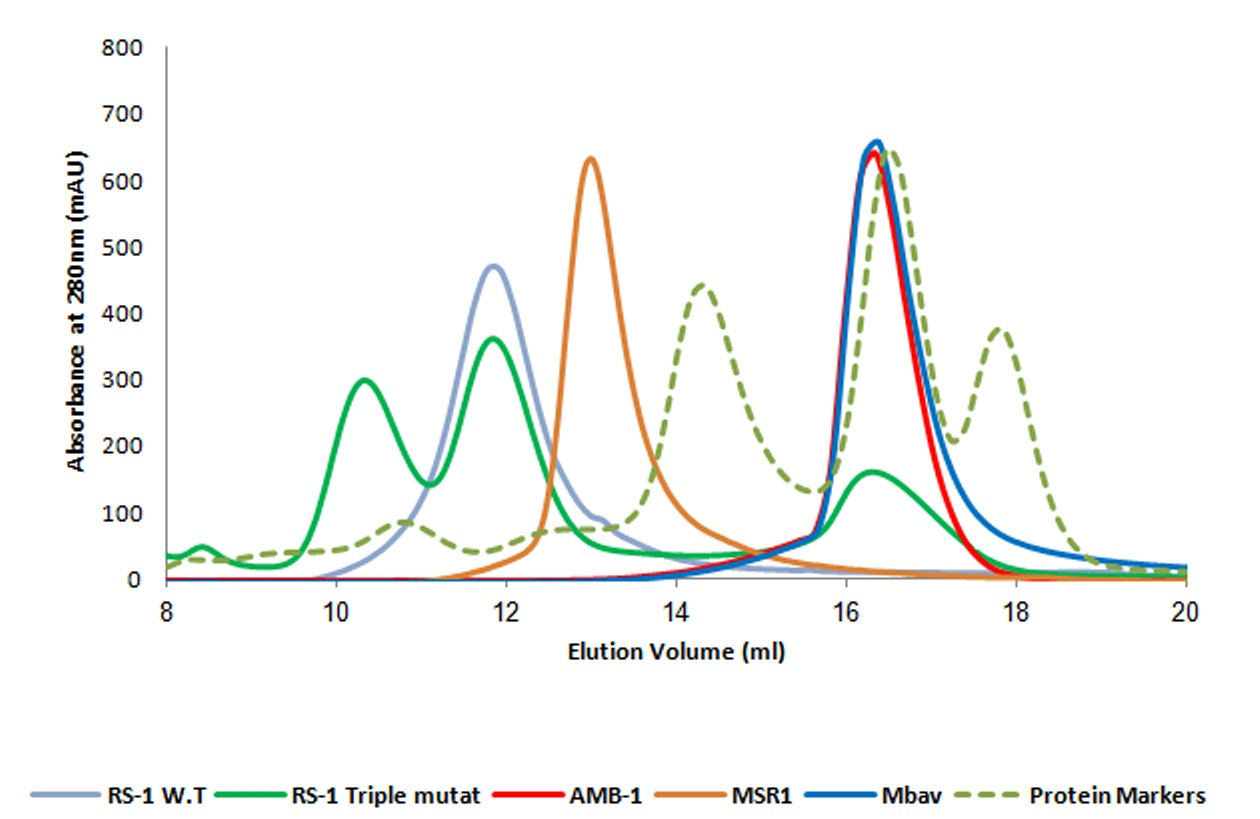

Supplement: S1 Fig — Elution profiles of MamAΔ41 triple mutant from Desulfovibrio magneticus (RS-1) and wild type MamAΔ41 from Desulfovibrio magneticus (RS-1), M. magneticum (AMB-1), M.gryphiswaldense (MSR-1) and Candidatus Magnetobacterium bavaricum (Mbav) colored in light blue, green, red, orange and blue, respectively. Wild type MamAΔ41 from RS-1 eluted in a volume corresponds to octamer (~192 kDa) whereas the triple mutated MamAΔ41 eluted in three separate peaks that correspond to a 13-monomer oligomer (~312 kDa), octamer (~192 kDa) and a monomer (~ 24 kDa). Both MamAΔ41 from AMB-1 and Mbav eluted at a volume corresponding to the monomer (20–22 kDa). MamAΔ41 from MSR-1 eluted at a volume typical of the trimer (~60 kDa). Dashed green line represents the elution profile of protein markers: Ferrritin (~440 kDa), Ovalbumin (~43 kDa), Carbonic Anhydrase (~29 kDa), Ribonuclease (~14 kDa). (TIF) [file pone.0133556.s001.tif]

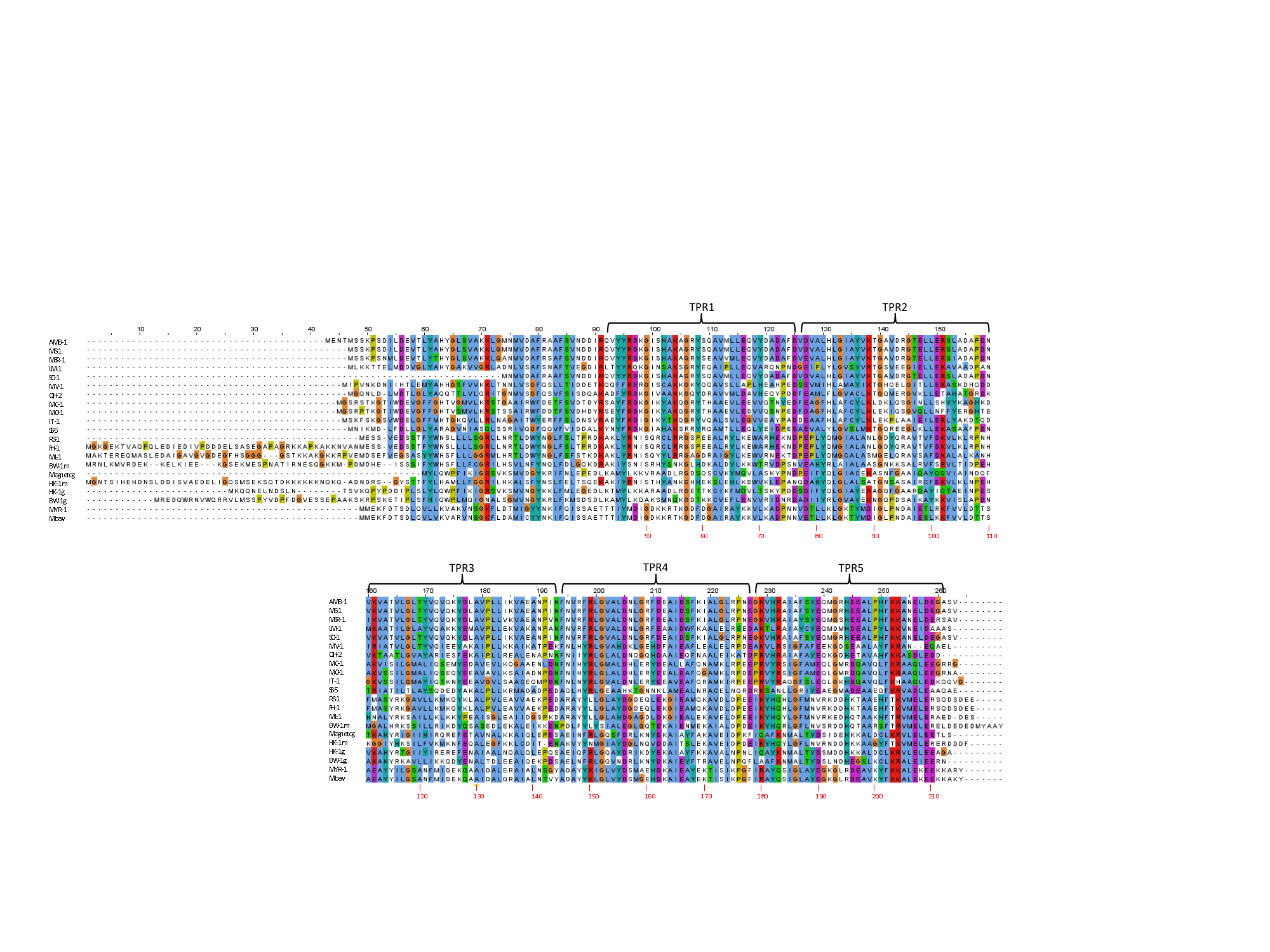

Supplement: S2 Fig — The MTB from Alphaproteobacteria class used in the analyses are: Magnetospirillum magnetotacticum (strain MS-1), Ms. magneticum (AMB-1), Ms. gryphiswaldense (MSR-1), strain SO-1, strain LM-1, Magnetovibrio blakemorei (MV-1), Magnetospira sp. QH-2, strain MO-1, Magnetofaba australis (IT-1) and Magnetococcus marinus (MC-1). Strain SS-5 from theGammaproteobacteria class is also used. From the Deltaproteobacteria class MTB used include the magnetotactic multicellular prokaryotes Ca. Magnetoglobus multicellularis (MMP) and strain HK-1, Ca. Desulfamplus magnetomortis (BW-1), Desulfovibrio magneticus (RS-1 and FH-1), and strain ML-1. Ca. Magnetobacterium bavaricum (Mbav) and strain MYR-1 of theNitrospirae phylum was also used. Red numbers at the bottom denote residue numbers specific for ArsTM. (TIF) [file pone.0133556.s002.tif]

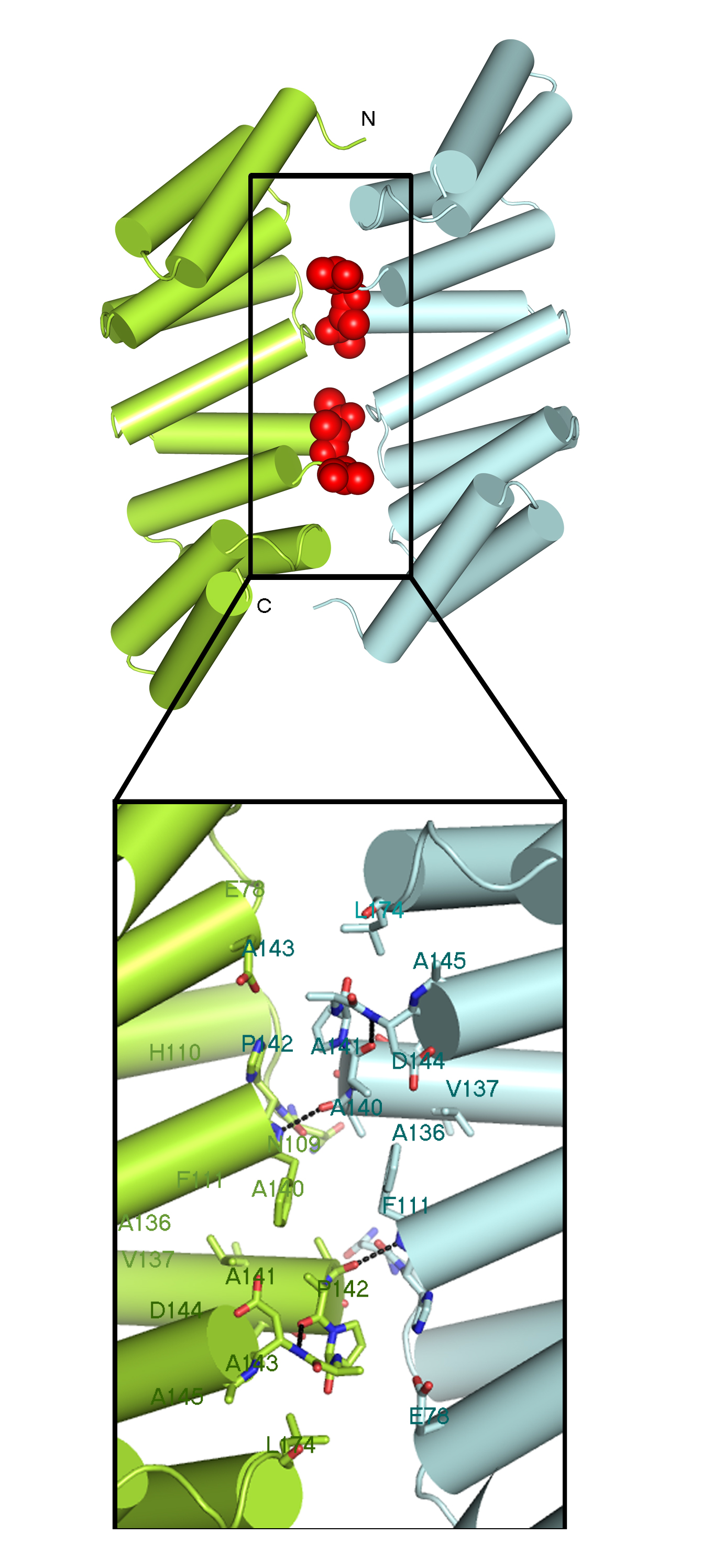

Supplement: S3 Fig — The triple mutated residues (E140A, K141A and E143A, highlighted as red spheres it the top view) are found in the centers of these interaction surfaces. (TIF) [file pone.0133556.s003.tif]

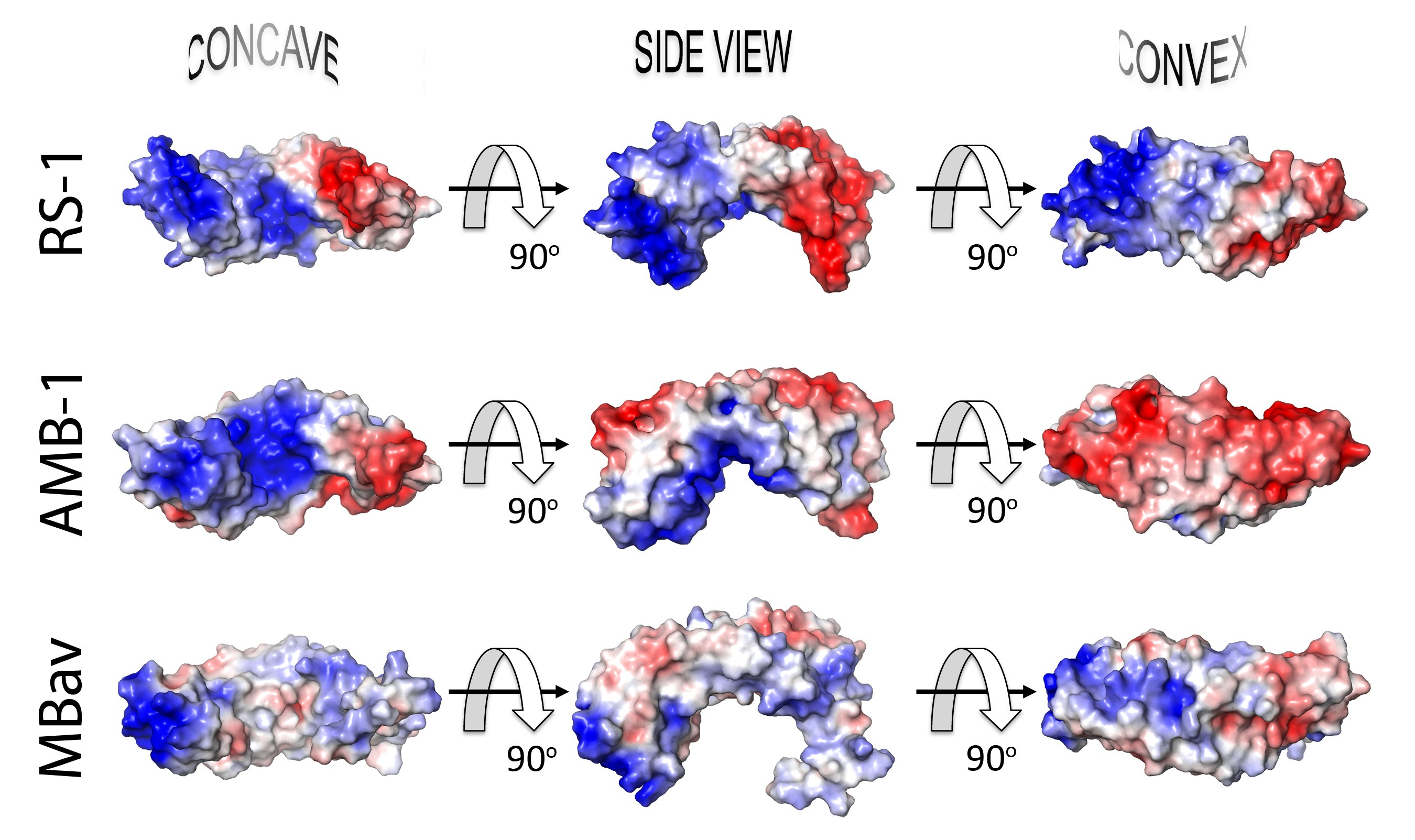

Supplement: S4 Fig — Surface charge comparison of MamAΔ41 structures, with blue and red colours representing regions of positive and negative electrostatic potential, respectively. The molecule is shown in three views, namely the concave surface, a side view and the convex surface, related by 90° rotations. The surface charge representation of ArsTM and MamAΔ41Mbav display a concave surface that is mainly positive and a convex surface that contains both positive and negative patches. The surface charge representation of MamAΔ41AMB-1 displays a concave surface that is extremely positive and a mainly negative convex surface. All electrostatic surfaces representations were produced with the APBS plug-in of PyMOL under the same contour levels. (TIF) [file pone.0133556.s004.tif]
